# High-stability solid solution perovskite (1-x) Bi_0.2_Sr_0.5_La_0.3_TiO_3_- xLaMnO_3_ (0.05≤ *×* ≤0.2) for wide-temperature NTC thermistors

**DOI:** 10.3389/fchem.2023.1275274

**Published:** 2023-09-29

**Authors:** Ting Liu, Guanghua Yang, Juan Ma, Huimin Zhang, Min Zhang, Aimin Chang

**Affiliations:** ^1^ State Key Laboratory of Chemistry and Utilization of Carbon Based Energy Resources, College of Chemistry, Xinjiang University, Urumqi, China; ^2^ Key Laboratory of Functional Materials and Devices for Special Environments of CAS, Xinjiang Key Laboratory of Electronic Information Materials and Devices, Xinjiang Technical Institute of Physics & Chemistry of CAS, Urumqi, China; ^3^ College of Physics, Xinjiang University, Urumqi, China

**Keywords:** negative temperature coefficient thermistor ceramics, perovskite-structured, oxygen vacancies, electronic properties, high-stability

## Abstract

The development of negative temperature coefficient (NTC) thermistor materials with a wide range of operating temperatures, high resistance (R), low thermal content (B) and good stability is significant for improving the overall performance of NTC thermistors. Traditional NTC thermistors materials are of the spinel, however, their practical applications are commonly limited to temperatures below approximately 200°C.In this study, it was found that a novel perovskite-structured solid solution (1-x)Bi_0.2_Sr_0.5_La_0.3_TiO_3_-xLaMnO_3_ (0.05 ≤ *×* ≤ 0.2) (BSLT-LM) showed good NTC performance from room temperature to high temperature (600°C) due to the stable structure at high temperatures. The ρ_25_, ρ_100_, ρ_600_ and B_25/100_, B_25/600_ constants of Bi_0.2_Sr_0.5_La_0.3_TiO_3_-0.1LaMnO_3_ NTC thermistors are approximately 1.76 × 10^8^ Ω cm, 1.13 × 10^7^ Ω cm, 9.89 × 10^2^ Ω cm, 4063.91 K, 5472.34 K, respectively. The electrical conductivity of these solid solution refers to the electronic transition between Mn^3+^ and Mn^4+^, and oxygen vacancies. These results demonstrate the tremendous potential of perovskite-structured (1-x) Bi_0.3_Sr_0.5_La_0.2_TiO_3_-xLaMnO_3_ thermistor ceramics with NTC performance.

## 1 Introduction

Accurate measurement and temperature control are very important in advanced industrial manufacturing. Negative temperature coefficient (NTC) thermistor-based thermally sensitive resistors are among the most popular temperature sensors (Feteira, 2009; Feteira and Reichmann, 2010). The advantageous features of these thermistors include their low cost, small size, excellent sensitivity, chemical and thermal stability, fast response time and high signal-to-noise ratio (Navi et al., 2012). However, the application of traditional spinel NTC thermistors is commonly limited to temperatures below approximately 200°C, because of the gradual collapse of the spinel structure and internal equilibrium of cation redistribution under a temperature of more than 200°C (Feteira, 2009; Yang et al., 2016; Alim et al., 2017).

High temperature NTC materials have received widespread attention corresponding with their growing need in the automobile exhaust testing, domestic appliances, and aerospace industries (Feltz and Pölzl, 2000; Houivet et al., 2004). Therefore, it is extremely urgent to explore new materials with stable structures and electrical characteristics at high and wide temperature ranges (0°C–600°C). In recent years, close attention has been paid to the application of perovskite in high-temperature NTC thermistors. These perovskite NTC thermistors show good performance due to their especial crystal structure. The perovskite structure (ABO_3_) consists of closed-packed AO_3_ layers, of which one-fourth of the octahedral interstitials are occupied by B cations (Bhalla et al., 2000; Feteira, 2009). The tetrahedra in the perovskite structure are empty, reducing its degree of complexity relative to spinel. Feltz (Alim et al., 2017) stated that the advantage of having anti-aging stability at higher temperature is closely related to the defined structure of perovskite-type compounds. The cations A and B of perovskite-type compounds are independent of temperature and are pinned at the lattice sites. Therefore, the cation migration hardly ever occurs between the dodecahedral A sites and the octahedrally coordinated smaller cations on B sites in perovskites, even at high temperatures (T. Liu et al., 2020). That is why perovskites exhibit stable electrical characteristics at high temperatures.

There are many work on new perovskite NTC thermistors. For example, MgAl_2_O_4_-YCr_0.5_Mn_0.5_O_3_ (Zhang et al., 2013) composite ceramics exhibit good NTC characteristics over a wide temperature range from 25°C to 1,000°C. La_2_O_3_-doped 0.6Y_2_O_3_-0.4YCr_0.5_Mn_0.5_O_3_ (Zhang et al.) composite ceramics show good NTC characteristics in a wide temperature range from 25°C to 1,000°C. The only limitation of these perovskite materials is their high sintering temperatures, that incurs additional costs and energy consumption. Preparing these ceramics requires harsh experimental environments, sintering temperatures close to 1700°C or requires special equipment such as SPS sintering and vacuum sintering. Other perovskite materials, such as BaTiO_3_ doped by BaBiO_3_ and La_2_O_3_(Ying et al., 2006) NTC thermistors ceramic can be sintered at 850°C for 4 h. Nevertheless, the temperature range of application is only from 25°C to 300°C. Besides this, there are only a few articles report the aging (resistance drift, ΔR/R) (Metz, 2000) of perovskite NTC materials at high temperatures.

Considering the importance of the preceding factors, we initiated the research on high-temperature NTC thermistors with stable structure and electrical characteristics at high temperatures. Recent work has found that perovskite-type (1-x) Bi_0.3_Sr_0.5_La_0.2_TiO_3_-xLaMnO_3_ (0.05 ≤ *×* ≤ 0.2) (BSLT-LM) solid solution have relatively low sintering temperature (1,150°C) and wide application temperature ranges (25°C–600°C) as NTC thermistor materials. BSLT-LM ceramics exhibited a good linear relationship between logarithm of electrical resistivity (ln ρ) and reciprocal of absolute temperature (1,000/T) at 25°C–600°C. NTC thermistors with high-resistance and low B-value NTC thermistors based on (1-x) Bi_0.2_Sr_0.5_La_0.3_TiO_3_-xLaMnO_3_ (0.05 ≤ *×* ≤ 0.2) materials were prepared by the sol-gel method. The electrical properties and microstructures were also evaluated. To date, this was the first confirmed BSLT-LM solid solution ceramic which exhibited good NTC characteristics in a wide temperature range (25°C–600°C), thereby providing a rational explanation for the conduction mechanism of BSLT-LM solid solution NTC ceramic thermistors.

## 2 Experimental

### 2.1 Sample preparation

Bi_0.2_Sr_0.5_La_0.3_TiO_3_ and LaMnO_3_ materials were prepared via the sol-gel technique. All reagents used in this work were of analytical grade. The synthesis procedure of Bi_0.2_Sr_0.5_La_0.3_TiO_3_ was as follows:stoichiometric amounts of lanthanum nitrate (La(NO_3_)_3_·6H_2_O) (99%, Aladdin) and strontium nitrate Sr(NO_3_)_3_ (99%, Aladdin) were dissolved in deionized water at a concentration of 0.5 mol/L (solution 1, concentration is 0.5 mol/L). Bismuth nitrate (Bi(NO_3_)_3_·5H_2_O) (99%, Aladdin) was completely dissolved in glycol (solution 2, the concentration is 0.5 mol/L). Tetrabuty-titanate Ti(OC_4_H_9_)_4_ (99%, Aladdin) was diluted in absolute ethanol and a few drops of acety-acetone were added to stabilize the titanium ions (solution 3, the concentration is 0.5 mol/L). Solutions 2 and 3 were dropwise added to solution 1. Then, citric acid (C_6_H_8_O_7_) was added as a chelating agent to the solution 1. The molar ratio of citric acid to total metal cations was 1.5:1. The mixed solution was stirred vigorously for 2 h to form a stoichiometric sol. A brown gel was formed after heating at 140°C for 8 h. The precursor was then calcined at 750°C for 2 h to obtain Bi_0.2_Sr_0.5_La_0.3_TiO_3_ powder. The synthesis procedure of LaMnO_3_ is same with above, but the gel was calcined at 850°C for 2 h to obtain the LaMnO_3_ powder.

The (1-x)Bi_0.2_Sr_0.5_La_0.3_TiO_3_-xLaMnO_3_ (x = 0.05, 0.1, 0.15, 0.2) samples were prepared by a solid-state method. (1-x)Bi_0.2_Sr_0.5_La_0.3_TiO_3_- xLaMnO_3_ precursor powders with different ratios (x = 0.05, 0.1, 0.15, 0.2) were thoroughly mixed, ground, and pre-calcined in air at 1,000°C for 2 h. After grinding, the powders were pressed into pellets (10 mm in diameter and 1 mm in height) under a pressure of 15 MPa followed by sintering in air at 1,150°C for 2 h. A silver paste was coated on both surfaces of the sintered pellets to function as electrodes.

### 2.2 Sample characterization

The crystal structure of the sintered powders was analyzed by X-ray diffraction (XRD) (Rigaku DMAX 2500, Japan) using Cu-Ka radiation at 40 kV and 25 mA. The microstructure of the sintered ceramic samples was investigated using scanning electron microscopy (SEM) (JEOL 5600, Japan) combined with energy-dispersive spectroscopy (EDS). The valence state of cations was analyzed by X-ray photoelectron spectroscopy (XPS, Thermo ESCALAB 250XI, United States). The binding energies obtained were calibrated based on the contaminated C1s peak (284.8 eV). The high resolution spectra of elements were fitted and deconvoluted using an Avantage software. To study the electrical properties, a conductive platinum paste with a thickness of 0.15 mm was spread on ceramic samples and dried at 850°C to function as electrodes. The electrical resistances of the BSLT-LM samples at temperatures range of 25–600°C in the atmosphere were characterized utilizing a digital multimeter (Agilent 34401A, United States), through two probe method. Additionally, the aging coefficient (ΔR/R_0_) of the 0.9Bi_0.2_Sr_0.5_La_0.3_TiO_3_- 0.1LaMnO_3_ ceramic sample was measured after heat treatment at 700°C for 200 h. The sintered density of the pellets have been characterized by Archimedean drainage method.

## 3 Results and discussion

**FIGURE 1:**
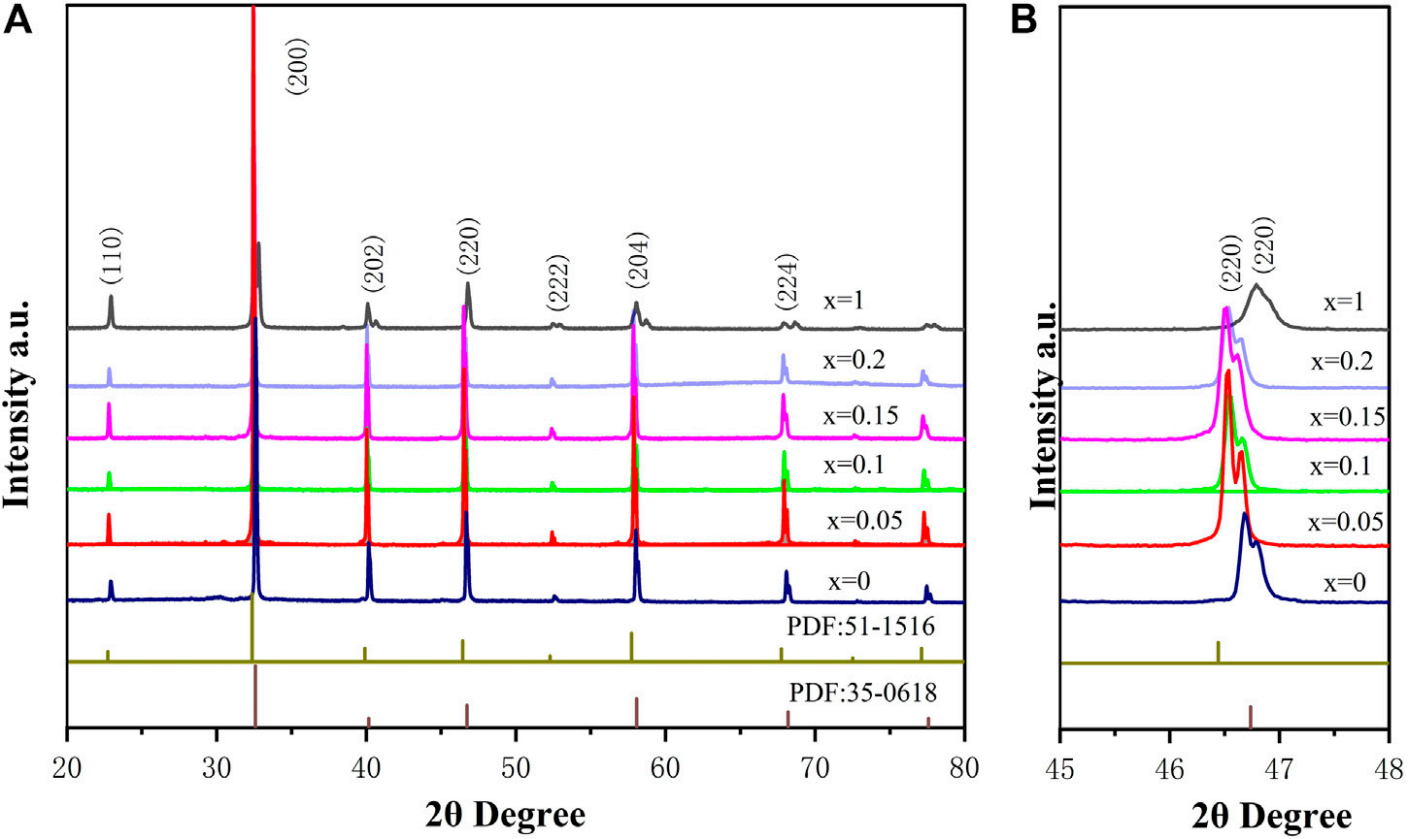
**(A)** XRD patterns of the (1-x) Bi_0.2_Sr_0.5_La_0.3_TiO_3_-xLaMnO_3_ (x = 0.05, 0.1, 0.15, 0.2) ceramics sintered at 1,150°C and **(B)** The detailed XRD patterns at room temperature.

XRD patterns of BSLT-LM solid solution ceramics sintered at 1,150°C are shown in Figure 1A. It can be seen from the XRD patterns that all samples possessed a pure perovskite phase with no detectable second phases. This indicates that LaMnO_3_ has completely diffused into the (1-x) Bi_0.2_Sr_0.5_La_0.3_TiO_3-x_LaMnO_3_ lattice to form solid solution (Navi et al., 2012; Huang et al., 2016). The main phase of (1-x) Bi_0.2_Sr_0.5_La_0.3_TiO_3_-xLaMnO_3_ (x = 0.05, 0.1, 0.15, 0.2) is the orthorhombic perovskite Bi_0.2_Sr_0.5_La_0.3_TiO_3_ phase (PDF#No.035-0618 card), which is isomorphic to SrTiO_3_ and both have a Pm-3m space group number of 221. In general, the presence of sharp and well-defined diffraction peaks indicates that the compound has good crystallinity. As can be seen from Figure 1B, the XRD patterns of partial enlarged view, solid solution BSLT-LM samples show peaks at the nearly same positions of LaMnO_3_. (Kim et al., 2007). For comparison, the patterns of Bi_0.2_Sr_0.5_La_0.3_TiO_3_ (x = 0, PDF#No.035-0618 card) and LaMnO_3_ (x = 1.0, PDF#No.051-1516 card) are provided. Besides, it should be noted that with the increase of the content of LaMnO_3_, the peaks corresponding to the Bi_0.2_Sr_0.5_La_0.3_TiO_3_ phase in the solid solution samples are slightly blunt and oriented toward a lower angle. The details are shown in Figure 1B. There are great possibilities of several reactions in solid solution, due to the ions have the similar ionic radius. The radius of ionic at A site of perovskite (ABO_3_) for Bi_0.2_Sr_0.5_La_0.3_TiO_3_ (x = 0): Bi^3+^ (0.103 nm), La^3+^ (0.1032 nm), Sr^2+^ (0.118 nm) and ions at B site: Ti^4+^ (0.0605 nm), respectively. The radius of ionic at A site of perovskite (ABO_3_) for LaMnO_3_ (x = 1) is La^3+^ (0.1032 nm), and ions at B site is Ti^4+^ (0.0605 nm), respectively. When LaMnO_3_ introduced to BSLT-LM, the Mn will occupy the B site (Ti site) and La occupy the A site. The radius of Mn^3+^ larger than Ti^4+^ maybe lead to the grain size increases and the diffraction angle gradual changes to low angle with x increase. For the better understanding the structural information, the XRD spectra of all the samples were refined and analyzed. The value of c/a ratio and lattice parameter for all of the BSLT-LM samples as given in Table 1. It can be seen that the value of c/a ratio of sintered ceramics gradually decrease with the increase of LaMnO_3_ content.

**TABLE 1:** Lattice parameter and the value of c/a ratio for the (1-x) Bi_0.2_Sr_0.5_La_0.3_TiO_3_-xLaMnO_3_ (x = 0.05, 0.1, 0.15, 0.2) ceramics.

x	Space groupe	Cell type	a (Å)	b (Å)	c (Å)	c/a
x = 0.2	Pm-3m	cubic	3.8962	3.8962	3.8925	0.9991
x = 0.15	Pm-3m	cubic	3.8961	3.8961	3.8965	1.0001
x = 0.1	Pm-3m	cubic	3.8959	3.8959	3.8968	1.0002
x = 0.05	Pm-3m	cubic	3.8949	3.8949	3.8978	1.0007

**FIGURE 2:**
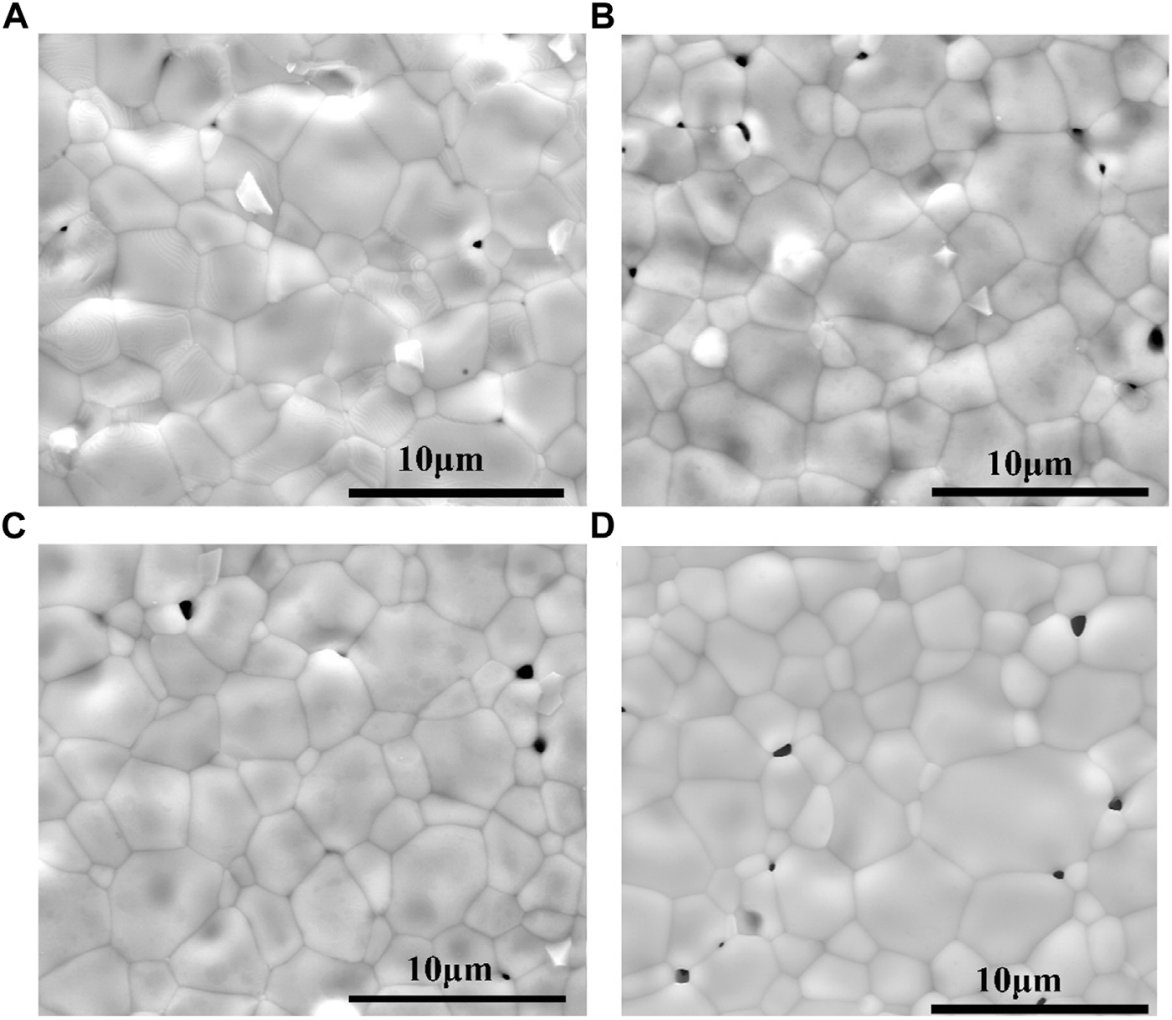
Representative SEM micrographs of (1-x)Bi_0.2_Sr_0.5_La_0.3_TiO_3_-xLaMnO_3_ ceramics surface: **(A)** x = 0.05, **(B)** x = 0.1, **(C)** x = 0.15, **(D)** x = 0.2 sintered at 1,150°C.

Figure 2 shows representative SEM micrographs obtained from the surfaces of (1-x)BSLT-xLM based ceramics sintered at 1,150°C, where x = 0.05, 0.1, 0.15, 0.2. All ceramics have dense and homogenous microstructures. This offers good reproducibility of electrical characteristics and improves the electrical properties of the ceramics. The grain size of the sample is in the range of 2–7 μm. Most of the pores in the sample were located at the grain boundaries. With the increase of the content of LaMnO_3_, the grain size became bigger and oversized grains even appeared when x = 0.2 (in Fig. d).

**FIGURE 3:**
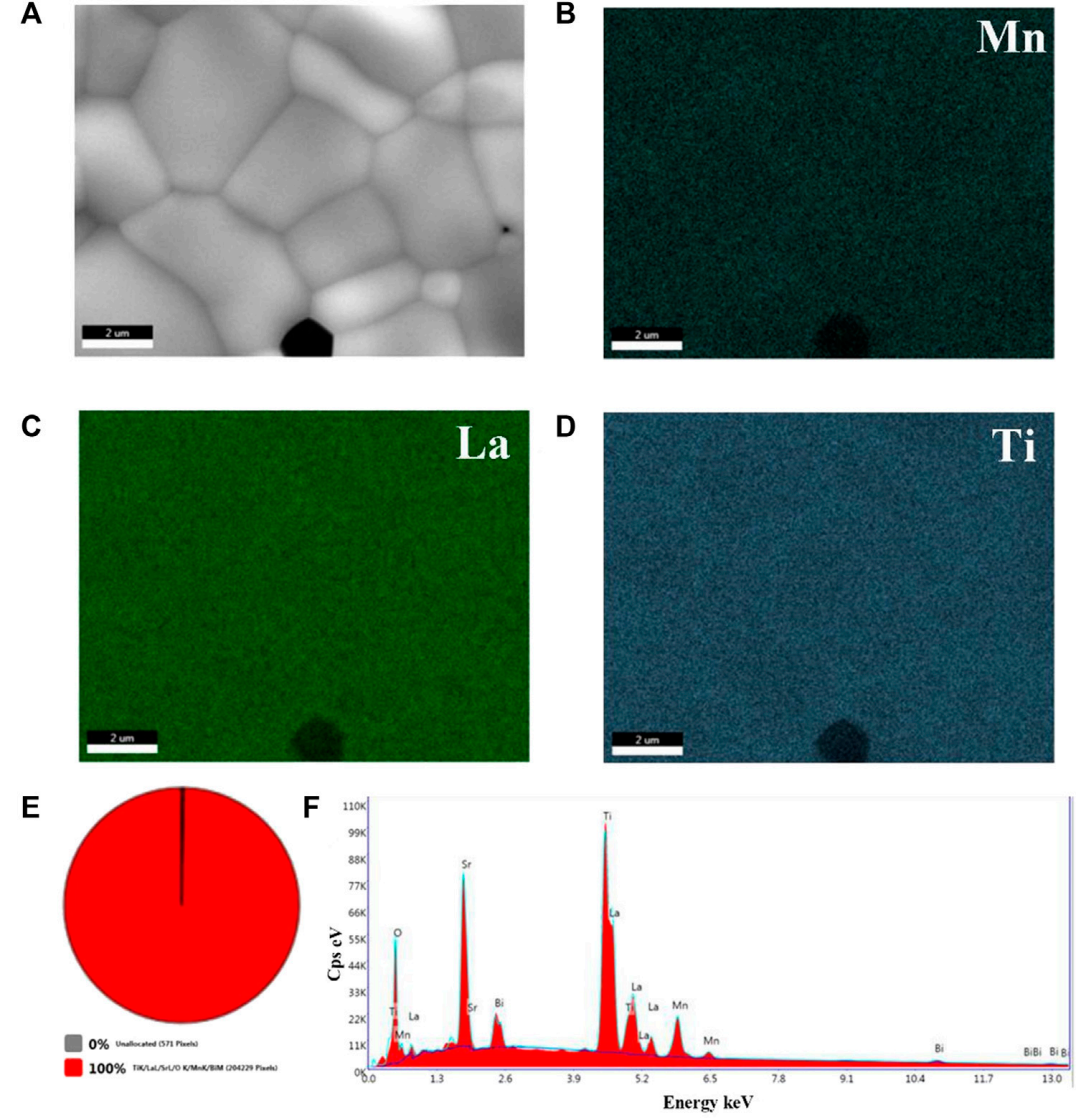
EDS elemental mapping results of the 0.9BSLT-0.1LM ceramics: **(A)** the SEM of 0.9BSLT-0.1LM EDS mapping analysis; **(B)**, **(C)**, **(D)** represent Mn, La, Ti atoms, respectively; **(E)** the phase distribution of 0.9BSLT-0.1LM EDS mapping analysis; and **(F)** the mapping analysis of elementary composition.

Here, EDS elemental mapping analysis was used to further analyze the composition distribution of 0.9BSLT-0.1LM ceramics (Figure 3). It can be found that Mn, La and Ti elements are distributed uniformly in the main phase. Figure 3E shows that there is only one solid solution phase in the 0.9BSLT-0.1LM ceramics. This indicates that LaMnO_3_ has completely diffused into the Bi_0.2_Sr_0.5_La_0.3_TiO_3_ lattice to form a solid solution, which is in accordance with the XRD results. The relative density of 0.9BSLT-0.1LM ceramic was 96%, which indicate that the ceramics prepared by the sol-gel method contribute to ceramic densification with lower porosity. Besides, many similar solid solution perovskite composite ceramics were applied in multiferroics, magnetoelectric and dielectrics field, such as BaTiO_3_-LaMnO_3_ composites (Kim et al., 2007; Dhak et al., 2016), 0.7BiFeO_3_–0.3BaTiO_3_–xMnO_2_ (X.-H. Liu et al., 2008), La_1-x_Sr_x_MnO_3_/BaTiO_3_ (Nguyen et al., 2017). Different from other composite materials, the high uniformity of those solid solution perovskites composites could improve the consistency and interchangeability of NTC thermistors.

**FIGURE 4:**
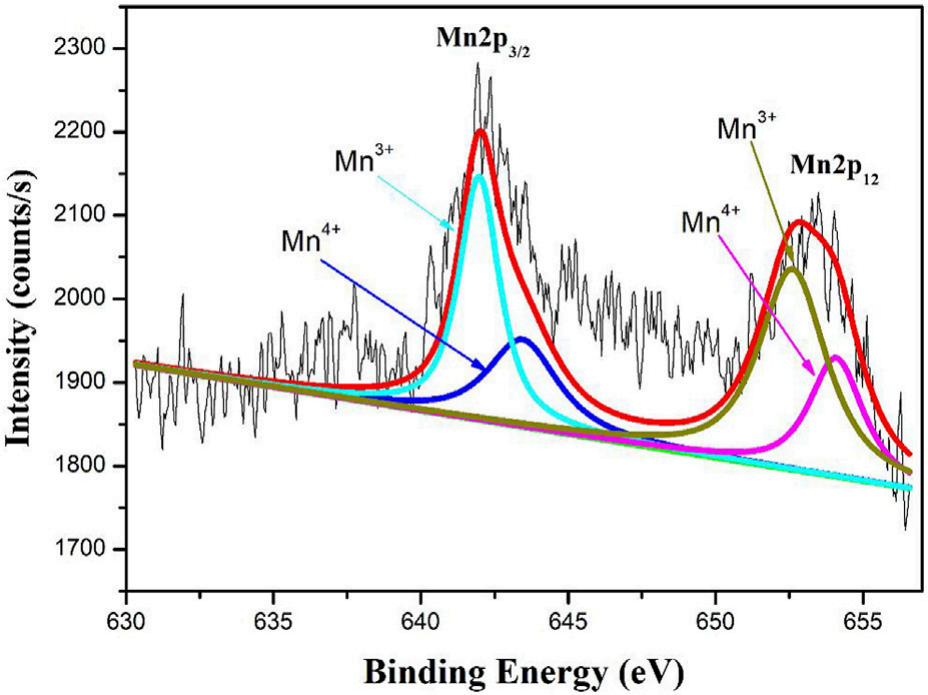
XPS spectra of Mn 2p regions of 0.9Bi_0.2_Sr_0.5_La_0.3_TiO_3_-0.1LaMnO_3_ ceramics.

The XPS spectra of Mn 2p regions of 0.9Bi_0.2_Sr_0.5_La_0.3_TiO_3_- 0.1LaMnO_3_ ceramic sample shown in Figure 4. According to the Gaussian-Lorentzian curve fitting, the peaks in the Mn 2p_3/2_ spectra can be split into two: a main peak at 641.1 eV can be conveniently resolved into the contribution of Mn^3+^ and a second peak at 642.5 eV to Mn^4+^(Zhang et al., 2014). These results indicate the presence of Mn^3+^and Mn^4+^ on lattice sites.

**FIGURE 5:**
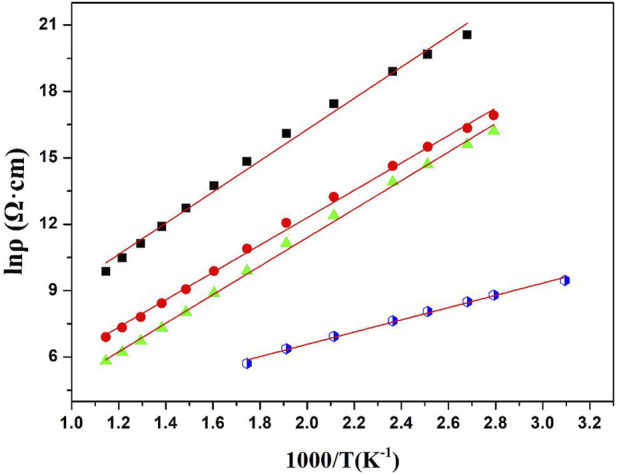
Shows the plots of the logarithms of the electrical resistivity, ln ρ, *versus* the reciprocal of the absolute temperature,1,000/T in the range of 25°C–600°C. a, b, c, d for the (1-x) Bi_0.2_Sr_0.5_La_0.3_TiO_3_-xLaMnO_3_ ceramics x = 0.05, 0.1, 0.15, and 0.2 respectively, which were sintered at a temperature of 1,150°C.

As can be seen from Figure 5, the NTC thermistors are stable and operate in a straight linear relationship between ln ρ and 1,000/T over a wide temperature range, which are the typical characteristics of NTC thermistors. Generally, the slope of the ln ρ *versus* 1,000/T curve is used to measure the activation energy of materials. The resistivity changes exponentially with temperature, which can be expressed by the following Arrhenius Eq. (1) (Bonet et al., 2016):

$$\rho =\rho_{0}\exp\left(\frac{B}{T}\right)$$

where ρ_0_ denotes the resistivity of the material at infinite temperature, T is the absolute temperature, and B is a constant which indicates the sensitivity to temperature excursions (sometimes called the coefficient of temperature sensitivity). The thermistor constant B has the magnitude of the activation energies, given by Eq. (2) (Kamlo et al., 2011):

$$B=\frac{Ea}{k}$$

where k is the Boltzmann constant, and Ea represents the activation energy of electrical conduction. The calculated B constants and activation energies as well as the resistivities at 25, 100°C and 300°C are listed in Table 2.

**TABLE 2:** Resistivities at 25°C, 100°C and 300°C, B_100/300_ constant, activation energy for the (1-x) Bi_0.2_Sr_0.5_La_0.3_TiO_3_-xLaMnO_3_ (x = 0.05, 0.1, 0.15, 0.2) ceramics samples.

X	ρ_25_ (Ω cm)	ρ_100_ (Ω·cm)	ρ_300_ (Ω·cm)	B_100/300_ (K)	Ea (eV)
0.05		586,259,000	2,798,496	5715.43 ± 4	0.492
0.1	175,669,780	11,347,578	53599.92	5726.62 ± 3	0.493
0.15	170,970,800	8,943,480	23868.39	6337.13 ± 6	0.549
0.2	17376.92	4586.74	334.05	2801.32 ± 1	0.241

**FIGURE 6:**
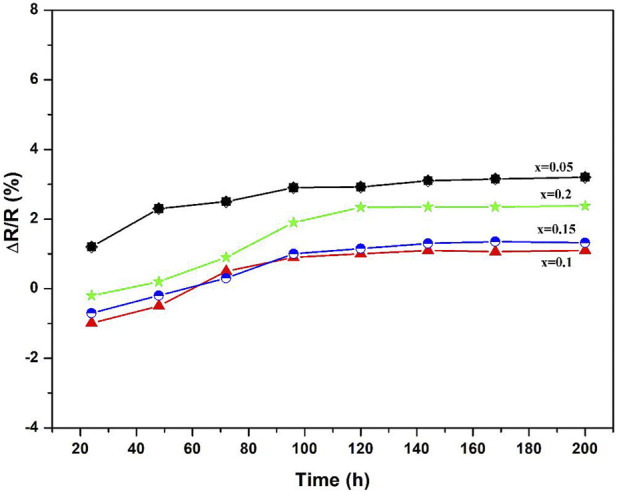
Aging coefficient of 0.9Bi_0.2_Sr_0.5_La_0.3_TiO_3_-0.1LaMnO_3_ ceramic sample as a function of aging time.

According to Table 2 the ρ_25_, ρ_100_, ρ_100_, B_100/300_ values and activation energy range of the (1-x)BSLT-xLM NTC thermistors with different compositions are 1.76×10^9^–1.74 × 10^4^ Ω cm, 5.86×10^8^–4.59 × 10^3^ Ω cm, 2.80×10^6^–3.34 × 10^2^ Ω cm, 2801–6337K, and 0.241–0.549eV, respectively. This means that the electrical properties of the (1-x) BSLT—x LM NTC thermistor ceramics can be adjusted to desired values by changing the LaMnO_3_ content. Additionally, the aging coefficient (ΔR/R_0_) of the 0.9Bi_0.2_Sr_0.5_La_0.3_TiO_3_-0.1LaMnO_3_ ceramic sample following heat treatment at 700°C for 200 h is shown in Figure 6. The plots show that the resistivity varies slightly within 1.8%. The good stability of BSLT-LM NTC thermistors at high temperatures is due to the special structure of their perovskite phase. The A and B cations of perovskite-type compounds are independent of temperature and are pinned at the lattice sites. Therefore, cation migration does not easily occur in perovskites even at high temperatures, which is why they exhibit stable electrical characteristics at high temperatures. Based upon the above results, the measured electrical performance values meet the requirements for use as industrial NTC thermistors (Yuan et al., 2015). It is therefore concluded that the partial composites of LaMnO_3_ for Bi_0.2_Sr_0.5_La_0.3_TiO_3_ are ideal for a wide range of practical applications as NTC thermistors.

**FIGURE 7:**
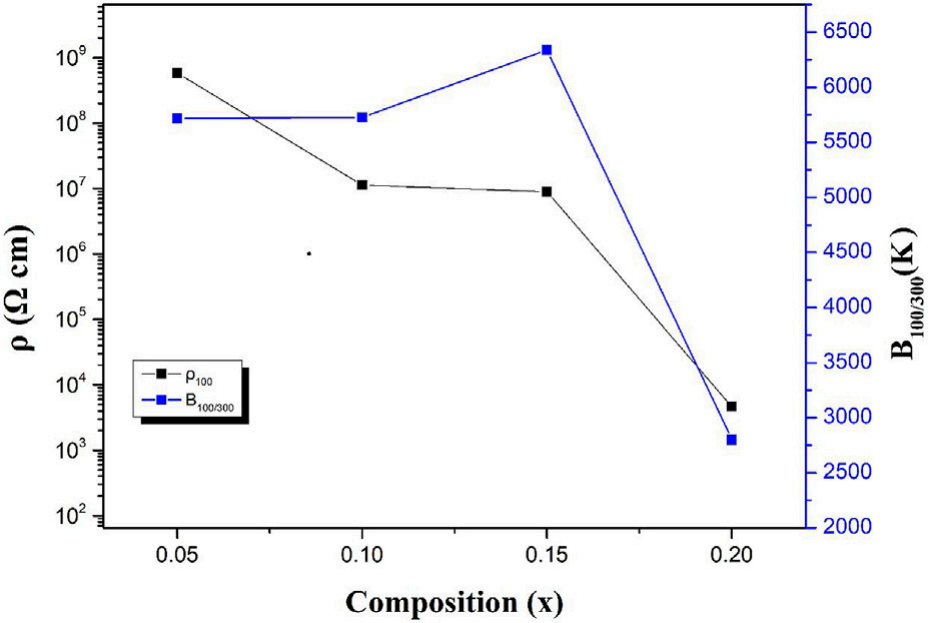
ρ_100_ and B constants as a function of the content x for (1-x) Bi_0.2_Sr_0.5_La_0.3_TiO_3_-xLaMnO_3_ ceramic samples.

The resistivity and B values of BSLT-LM samples are plotted as a function of composition “x” in Figure 7. It can be seen the ρ_100_ decreases as the content of LaMnO_3_ increases, while B_100/300_ increases first and then decreases. This result can be explained by the electrical conductivity in these ceramics. The electrical conductivities in these compounds refer to the oxygen-vacancies and the electronic transitions between Mn^3+^ and Mn^4+^: 1) It is well known that Bi-based materials exhibit higher oxygen-vacancies due to the lack of Bi. In addition, their weak Bi–O bonds and 6s two lone pair electrons provide a low diffusion barrier for ion migration. The oxygen vacancies in BSLT-LM ceramics were caused by the loss of a small amount of Bi_2_O_3_ during the sintering process (Li et al., 2014; Cho et al., 2017): ${2\text{Bi}}_{\text{Bi}}^{\times }+{3O}_{O}^{\times }\;\to \;{2V}_{\text{Bi}}^{\cdots }+{3V}_{O}^{∙∙}+{\text{Bi}}_{2}O_{3}$ (Eq. 3); 2) Due to the versatility of the valence states of Mn, Mn^3+^ was partially oxidized to yield Mn^4+^ during sintering in air. XPS results also confirm both the Mn^3+^ and Mn^4+^ in the BSLT-LM ceramics. The electrons in Mn^3+^ ions hop to Mn^4+^ through the following reaction: Mn^3+^-Mn^4+^ +e′ (Zhang et al., 2014). This leads to a higher electron concentration with a consequent lowering of the energy barrier for polaron hopping. Besides, LaMnO_3_ as low resistance phase doped in BSLT-LM solid solution ceramics. With the increase of LaMnO_3_ content, the amount of charge carriers increasing which are responsible for hopping and conductivity, and the resistivity of BSLT-LM ceramics decrease. When x = 0.15, there are a critical value of the thermal constant B_100/300_ to the hopping conduction based on oxygen-vacancy migration combined with polaron hopping, so the B_100/300_ increases first and then decreases.

## 4 Conclusion

In current study, a novel solid solution perovskite Bi_0.2_Sr_0.5_La_0.3_TiO_3_-xLaMnO_3_ samples were prepared by Pechini wet chemistry method and conventional sintering at 1,150°C. The sintered ceramic samples are solid solution perovskite phase confirmed by XRD and phase characterization analysis. The results of electrical characterization for the first time confirm that the Bi_0.2_Sr_0.5_La_0.3_TiO_3_-0.1LaMnO_3_ ceramics exhibit good negative temperature coefficient behavior over a wide temperature range (25°C–600°C). The ρ_25_, ρ_100_, ρ_600_ and B_25/100_, B_25/600_, constants of Bi_0.2_Sr_0.5_La_0.3_TiO_3_-0.1LaMnO_3_ NTC thermistors are approximately 1.76 × 10^8^ Ω cm, 1.13 × 10^7^ Ω cm, 9.89 × 10^2^ Ω cm, 4063.91 K, 5472.34 K, respectively. The aging drift rate is below 1.8%. The electrical properties of the (1-x) BSLT-xLM NTC thermistor ceramics can be adjusted to the desired values by changing the LaMnO_3_ content. The measured electrical performance values meet the requirements for use as industrial NTC thermistors. The electrical conductivities of these ceramics refer to oxygen-vacancy migration and small polaron hopping from the electronic transitions between Mn^3+^ and Mn^4+^.

## Data Availability

The original contributions presented in the study are included in the article/supplementary material, further inquiries can be directed to the corresponding authors.
